# What impact do chronic disease self-management support interventions have on health inequity gaps related to socioeconomic status: a systematic review

**DOI:** 10.1186/s12913-020-5010-4

**Published:** 2020-02-27

**Authors:** Ruth Hardman, Stephen Begg, Evelien Spelten

**Affiliations:** 1 La Trobe University Rural Health School, 471 Benetook Avenue, Mildura, Victoria 3500 Australia; 2 Sunraysia Community Health Services, 137 Thirteenth Street, Mildura, Victoria 3500 Australia; 3 La Trobe Rural Health School, La Trobe University, PO Box 199, Bendigo, Victoria 3552 Australia

**Keywords:** Self-management, Socioeconomic status, Health inequity, Patient capacity, Chronic disease

## Abstract

**Background:**

The social gradient in chronic disease (CD) is well-documented, and the ability to effectively self-manage is crucial to reducing morbidity and mortality from CD. This systematic review aimed to assess the moderating effect of socioeconomic status on self-management support (SMS) interventions in relation to participation, retention and post-intervention outcomes.

**Methods:**

Six databases were searched for studies of any design published until December 2018. Eligible studies reported on outcomes from SMS interventions for adults with chronic disease, where socioeconomic status was recorded and a between-groups comparison on SES was made. Possible outcomes were participation rates, retention rates and clinical or behavioural post-intervention results.

**Results:**

Nineteen studies were retrieved, including five studies on participation, five on attrition and nine studies reporting on outcomes following SMS intervention. All participation studies reported reduced engagement in low SES cohorts. Studies assessing retention and post-intervention outcomes had variable results, related to the diversity of interventions. A reduction in health disparity was seen in longer interventions that were individually tailored. Most studies did not provide a theoretical justification for the intervention being investigated, although four studies referred to Bandura’s concept of self-efficacy.

**Conclusions:**

The limited research suggests that socioeconomic status does moderate the efficacy of SMS interventions, such that without careful tailoring and direct targeting of barriers to self-management, SMS may exacerbate the social gradient in chronic disease outcomes. Screening for patient disadvantage or workload, rather than simply recording SES, may increase the chances of tailored interventions being directed to those most likely to benefit from them. Future interventions for low SES populations should consider focussing more on treatment burden and patient capacity.

**Trial registration:**

PROSPERO registration CRD42019124760. Registration date 17/4/19.

## Background

Chronic health conditions are increasingly common, with some population groups, such as those of lower socioeconomic status (SES) having both a greater incidence of chronic disease and a poorer prognosis [1–3]. The long-term nature of these conditions means that the patient is largely responsible for day-to-day disease management [4, 5] and since many chronic conditions are lifestyle-related [6], the quality of patient self-management is important. Self-management support (SMS) approaches have been developed to give people the skills to more effectively manage their health. These interventions involve both education and behaviour change strategies to address the medical, physical, emotional and social challenges associated with CD, aiming to help the person adapt to their changed circumstances whilst still leading a meaningful life [4, 5, 7].

Although SMS interventions are now widespread, outcomes have been mixed, with the benefits being limited to short-term improvements in psychological variables such as self-efficacy, rather than sustained clinical or behavioural changes [4, 6, 8, 9]. Most SMS interventions are theoretically grounded in Bandura’s concept of self-efficacy [5] and utilise specific techniques to enhance self-efficacy [5–7, 10, 11]. Self-efficacy theory refers to an individual’s belief or confidence in their capacity to undertake tasks or achieve goals, which can translate into health behaviour change and by implication, improved health status [4, 5].

Persisting questions remain, however, about the effectiveness of SMS in low SES and other disadvantaged groups. The original SMS trials were conducted in self-selected, higher SES populations [4, 6, 10] and studies in disadvantaged populations have reported poorer outcomes and lower levels of adherence [12, 13]. Several writers have theorised that the individual patient focus of SMS limits its effectiveness in these groups. By prioritising individual self-efficacy and activation, the potential barriers to self-management within the patient’s wider social context (e.g. literacy, resources, social supports) are ignored [6, 10, 11, 14, 15]. Although the dominant role of the social determinants of health is acknowledged in CD epidemiology, their influence on treatment engagement is rarely addressed [15].

Effective chronic disease (CD) management should include both an improvement in overall population health and a reduction in health inequities [16–18]. An intervention that appears more effective in a better-off population may widen the disparity gap, and there are strong suggestions that individually-focussed ‘downstream’ interventions, such as SMS, can increase disparity [17, 19, 20]. Specific targeting of disadvantaged groups is one way to deal with inequity, and tailored SMS approaches for these groups have been trialled, but systematic reviews have shown inconsistent and dose-dependent benefits [13, 21]. In addition, such interventions may have positive outcomes, but still not address the disparity gap [18].

Although there are suggestions that SMS interventions may be less effective in low SES groups, this can only be determined by comparing SMS outcomes between more and less advantaged groups. There have been no previous reviews on this topic, despite many researchers stressing the importance of addressing and quantifying the equity gap in CD [18–20, 22, 23]. This is partly due to statistical challenges, since the evidence will emerge from subgroup analyses [19, 24, 25]. However, given the strong connection between the social determinants of health and health outcomes, subgroup analyses need not be post-hoc data dredging but can be planned and valid approaches to answering these questions [26–28].

This review aims to examine studies that have looked at differences between socioeconomic groups undergoing SMS interventions, in order to answer the following questions: 1. Is there evidence that SES influences participation rates in SMS interventions? 2. Is there evidence that SES influences rates of retention or dropout from SMS interventions? 3: Is there evidence that SES affects clinical, behavioural or other specified outcomes following SMS interventions?

## Methods

### Search strategy and data abstraction

We conducted a systematic review of the literature using the PRISMA reporting guidelines [29] to structure the report. We searched for full-text articles in English to December 2018 in the following databases: Cochrane database; PubMed; Cinahl; Embase; Proquest and Psychinfo. The search terms covered the following areas, using MeSH terms and synonyms: [1] Chronic condition, including diabetes, cardiovascular disease, musculoskeletal conditions and chronic pulmonary disease [2]; Self-management [3]; Socio-economic status, including associated terms such as inequity, disparity, ‘vulnerable groups’; and [4] Terms related to outcomes, efficacy, retention or participation. The PubMed search strategy is available in Additional file 1. No date filter was employed in order to obtain the widest possible search. In the course of the search thirteen related systematic reviews were located and their references were screened resulting in seven additional papers.

### Inclusion criteria

Inclusion and exclusion criteria are outlined in Table 1. We looked for four main chronic conditions: cardiovascular disease (CVD), musculoskeletal conditions (MSK), pulmonary disease (COPD) and diabetes. All these conditions contribute significantly to the burden of disease and share many common risk factors. We included studies of co/multimorbidity since this is representative of the CD population. A decision was made to focus only on socio-economic status (SES), which has well-documented and consistent effects on chronic disease, rather than on other WHO PROGRESS+ factors such as gender and ethnicity, which can vary between countries [19]. All studies needed to provide a comparison between a less and more advantaged group, based on income, education or socioeconomic area. Comparisons based on literacy or ethnicity were only included if there was a quantifiable relationship between these variables and other SES measures. As well as post-intervention outcomes such as behavioural or clinical changes, outcomes related to participation and dropout were included to fully capture potential areas of disparity. Study designs could include randomised controlled trials with subgroup analyses, pre-post designs, cross-sectional or longitudinal data analyses.

**Table 1:** Inclusion/exclusion criteria

PICO	Inclusion Criteria	Exclusion Criteria
Population	Over 18 years	
	Diagnosed with diabetes, COPD, cardiovascular disease, chronic musculoskeletal pain and any additional comorbidities	At-risk patients (e.g. prediabetes)
	SES described in terms of education, income, area or occupation.	‘Disadvantaged’ (e.g. ethnic minority) population without quantifiable reference to SES.
Intervention	Includes a self-management support intervention incorporating at least 3 recognised elements of SM [7]	Single-component SMS intervention (e.g. education, medication adherence only).
Comparison	Includes analysis of whether the response to the intervention differs according to SES.	No measurement of SES disparity in reporting of outcomes.
Outcome	Reporting of outcomes which may be clinical, behavioural, psychosocial or related to participation/attrition.	

### Search outcomes

**Fig. 1:**
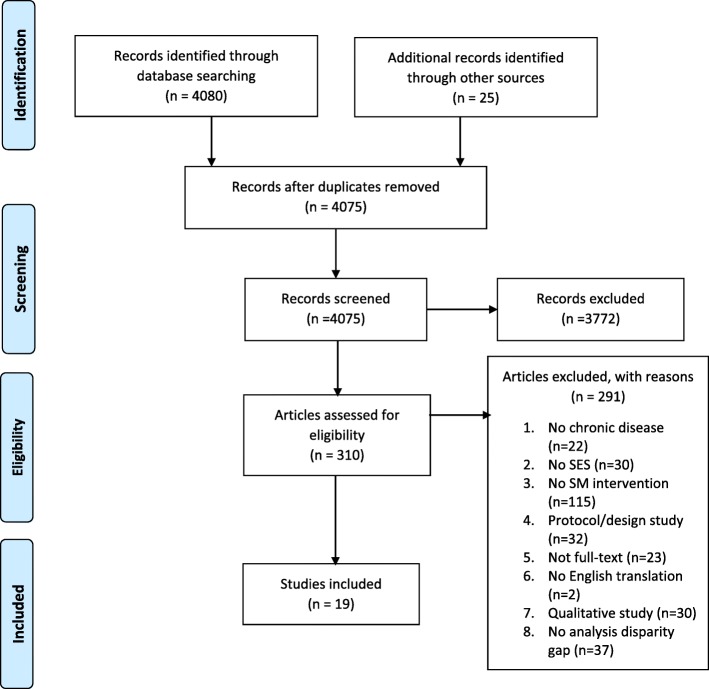
PRISMA diagram

Title and abstract screening reduced the number of papers to 310. Articles were excluded according to the criteria outlined in Table 1. Common reasons for exclusion were no SMS intervention (e.g. studies of self-care or adherence behaviours); SES not quantified, and no measurement of SES disparity. A full list of reasons for exclusion of the 291 full-text articles is available in Additional file 2. Figure 1 illustrates the search process undertaken. One reviewer (RH) completed the initial search and a second reviewer (ES) independently assessed the final papers to ensure agreement on inclusion criteria. Nineteen studies were included in the review.

### Data abstraction

The data was summarised on the setting, study design, type of CD, sample size, description of intervention and control, outcomes or variables measured, follow-up time, results and study quality (Table 2 and Table 3). Table 4 and Table 5 summarises data related specifically to SES and disparity, including the theory behind the SM intervention (or study question for participation/attrition studies), intervention description, SES adaptations made, SES status of population, results in relation to SES, dropout rates and overall impact on SES disparity. Related papers were retrieved to provide additional data about the population or intervention as needed [31–38].

**Table 2:** Studies examining disparities in outcomes following SM interventions, stratified by quality^1^

Author^1^	Country and setting	Study design	Chronic Disease	Sample size	Intervention description, healthcare providers (HCPs), SM Components^2^	Control	Outcomes measured	Follow up	Results	Quality rating
Rothman 2004 (Rothman 2005)	USA Public primary care clinics	RCT with subgroup analysis	Diabetes	217	Individual Phone and face-to-face SMS over 12/12 Pharmacist and nurse 1,2,3,5,6,7	Single session with pharmacist	Hb A1c and blood pressure	12/12	HbA1c improved significantly from baseline for both I/C. For higher literacy participants group there was no difference between I/C but those with low literacy had a HbA1c change of −1.4% (adjusted), CI −2.3 to − 0.6%, *p* < 0.001, favouring intervention. BP improved in intervention group regardless of literacy, *p* = 0.006	JBI 11/12 S/O 11/11
DeWalt 2012 (DeWalt 2006)	USA Hospital clinics	RCT with subgroup analysis	Chronic heart failure (HF)	605	Individual Education session then phone support for 12/12 Health educators 1,2,3,5,7,8	Single 1–1 education session	All-cause hospitalisation, death, HF hospital admission, HFQOL	12/12	In low-literacy participants adjusted incident rate ratio (IRR) was 0.73 for all-cause hospitalisation and death and 0.48 for HF hospitalisation, favouring intervention; IRR for high literacy was 1.16 for all-cause and 1.34 for HF hospitalisation, favouring control.	JBI 10/12 S/O 11/11
Bosma 2011 (Lamers 2010)	Netherlands Public primary care clinics	RCT with subgroup analysis	Diabetes or COPD with mild to moderate depression.	361	Individual Home-based CBT and SMS for 6/52 Nurses 1,3,4,6,7,8	Usual GP care	Depression primary outcome (Beck Depression Inventory); also health-related quality of life (QOL); control beliefs (mastery); self-efficacy.	9/12	Interaction between education level was significant (*p* < 0.05) or nearing significance (*p* < 0.10) at 3 and 9 months for all outcomes with no benefit for low educated. Clinically significant (> 50% improvement) in depression at 9 months for high educated only.	JBI 11/12 S/O 9/11
Moskowitz 2013 (Thom 2013)	USA Public primary care clinics	RCT with subgroup analysis	Diabetes	299	Individual Phone and face-to-face peer support over 6/12 Peer health coaches 1,3,4,5,6,8	Usual GP care	HbA1c	6/12	HbA1C reduced by 1.07% (intervention) vs 0.3% (control), *p* = 0.01. HbA1c decrease was predicted by SM ability and medication adherence. Those with low SM ability benefited most; ethnicity and education did not differentially affect the outcome.	JBI 10/12 S/O 9/11
Powell 2010	USA Hospital clinics	RCT with subgroup analysis	Heart failure	902	Group SMS classes over 12/12 Health professionals 1,2,3,4,5,6	Education sheets plus phone follow-up	Death/HF hospitalisation, medication adherence, salt intake, SM ability, cardiac QOL, SF 36, depression.	2.5 years	Depression, self-efficacy and salt intake improved in both intervention and control groups. Low income participants in the control group had a non-significant (*p* = 0.056) trend to earlier cardiac event (death/hospitalisation).	JBI 11/12 S/O 6/11
Smeulders 2010 (Smeulders 2006)	Netherlands Hospital clinics	RCT with subgroup analysis	Chronic heart failure	317	Group Stanford CDSMP for 6/52 Nurse and peer leader 1,2,3,4,5,6,7,8	Usual care	Cardiac QOL (Kansas City Cardiomyopathy Questionnaire)	12/12	Short-term improvement in cardiac QOL in intervention group but not at 6 or 12/12. Lower educated patients improved more than higher educated (*p* = 0.018) throughout the follow-up period.	JBI 10/12 S/O 7/10
Jonker 2012	Netherlands Elderly daycare facility	RCT with subgroup analysis	Frail elderly; unspecified chronic disease (mean of 2 CDs)	63 (intervention group)	Group Stanford CDSMP for 6/52 Nurses 1,2,3,4,5,6,7,8	Waitlist	Depression, valuation of life, control beliefs (mastery); self-efficacy, cognitive function.	6/12	Mastery (*p* = 0.01) and Depression (*p* = 0.05) scores improved from baseline in the intervention group at 6/12 (small effect size); subgroup analysis showed improvements in mastery (*p* < 0.05) were limited to the lower educated and those with better cognitive function.	JBI 6/12 S/O 5/11
Nour 2006	Canada Public community health centres	RCT with subgroup analysis	Arthritis and housebound	58 (intervention group)	Individual Home-based CBT and SMS for 8/52 Allied HCPs 1,3,4,5,6,8	Waitlist	Health behaviour changes, arthritis score, pain/fatigue scores, mastery, depression, self-efficacy.	8/52	Increased frequency of exercise (*p* < 0.001) and relaxation (*p* = 0.05) in intervention group but not for those with depression or perceived low SES.	JBI 5/12 S/O 5/11
Govil 2009	USA Insurance funded clinics	Cohort study	Cardiovascular disease	785	Individual and group 3/12 lifestyle programme Range of HCPs 1,3,4,5,6,7,8	None	Blood pressure, lipids, exercise tolerance, BMI, depression, adherence.	3/12	Outcomes improved significantly *p* < 0.05 across all education and income levels. Adherence and attendance similar across all groups. Baseline measures were significantly lower in low educated.	JBI 9/11

^1^Studies listed in order of quality as measured by Johanna Briggs Institute (JBI) criteria [30] and Sun/Oxman (S/O) subgroup analysis (for RCTs) criteria [27, 28]. RCTs listed first, followed by cohort studies.

^2^Includes additional studies from the same research group where supplementary information was obtained.

^3^Numbers correspond to the key components of self-management interventions as listed by Barlow et al. (Barlow): 1. Information 2. Drug management 3. Symptom management 4. Psychological management 5. Lifestyle management 6. Social support 7. Communication 8. Other (action planning, goal setting, decision making, problem solving, spirituality).

**Table 3:** Studies examining disparities in participation or attrition from SM interventions, stratified by quality^1^.

Author^2^	Country and setting	Study Design	Chronic Disease	Sample size	Intervention	Variables measured	Results	Quality rating
Poduval 2018 (Murray 2017)	UK Urban public primary care practices	Subgroup analysis of RCT	Diabetes	299 (intervention group)	Comparing 2 internet SM programmes +/− support Predictors of use	Gender, age, ethnicity, education.	No difference in frequency of programme use or registration according to any demographic predictors. User characteristics were reflective of the overall target population of the area.	JBI 12/12 S/O 10/11
Thorn 2011 (Day 2010)	USA Rural public primary care practices	Subgroup analysis of RCT	Chronic pain	109	Low-literacy pain SM (education and CBT) groups. Drop-out predictors	Demographics, literacy, pain catastrophising, disability, depression, QOL, pain intensity/interference.	Dropout before programme started was associated with low education (*p* < 0.02), low literacy (*p* < 0.05) and catastrophising (*p* < 0.01); failure to complete programme associated with income (under/over $13,000 – *p* < 0.01) and low education (*p* < 0.02).	JBI 12/12 S/O 9/11
Dattalo 2012 (Boult 2011)	USA Primary care (both insured and public patients)	Subgroup analysis of RCT	Multimorbid chronic disease	241	Stanford CDSMP Completion predictors	Demographics, health status, health activities, patient activation, patient-rated quality of care.	22.8% of eligible adults completed (attended at least 5 of 6 sessions). Attendance was associated with dissatisfaction with GP (OR = 2.8) and having higher SF-36 physical health scores (OR = 2.3). Age, sex, education, race and SES were not significant.	JBI 11/12 S/O 5/11
Cauch-Dudek 2014	Canada National database analysis	Cohort	Diabetes –first 8/12 post diagnosis	46,553	Any type of DSME Participation predictors	Age, sex, immigrant status, comorbidity, mental illness, rural residence, SES	22% of people attended DSME within 8/12 of diagnosis. Non- attendance was associated with older age, lower SES, recent immigration or physical/mental health comorbidity (all *p* < 0.001).	JBI 10/11
Adjei-Boakye 2018	USA National telephone survey	Cross-sectional	Diabetes	84,179	Any type of diabetes SM education (DSME) Participation predictors	Race, education, marital status, income, sex, health insurance, BMI, insulin use, self-care behaviour.	53.7% reported attending DSME, with attendance less likely amongst men (adjusted OR = 0.85), Hispanics (aOR = 0.81), high school only (aOR = 0.71) or less than high school educated (aOR = 0.51), income <$15,000 (aOR = 0.70) or < $25,000 (aOR = 0.81) and the uninsured (aOR = 0.87). Attending DSME was significantly associated with adherence to SM behaviours.	JBI 8/8
Glasgow 2018	USA Database analysis (health insurance organisation)	Cross-sectional	Diabetes	2603	Internet SM programme Participation predictors	Socio-demographics, reason for declining service, HbA1c BP, BMI, lipids, SF36, ADL, number of comorbidities	Participants were likely to be younger (*p* = 0.041); not Latino (*p* = 0.002); earning >$30,000 (*p* < 0.0001), greater than high school educated (*p* < 0.0001), non-smokers (*p* < 0.0001) with lower blood pressure (*p* = 0.028). Self-selected participants were the most likely to be white, better educated and healthier.	JBI 8/8
Horrell 2017	USA National database analysis	Cross-sectional	Multimorbid chronic disease	19,365	Stanford CDSMP Participation and completion predictors	Enrolment and completion of CDSMP compared to high/low SES area	83.6% of participants lived in the least impoverished areas (< 25% of population below poverty line) and 0.3% of participants lived in the most impoverished areas (> 50% below poverty line). SE area was significantly correlated with ethnicity and education level. Course completion was not associated with SES – poorer people had a higher (but non-significant) completion rate.	JBI 8/8
Hardman 2018	Australia Rural community health centre	Cross-sectional	Chronic pain	186	Tailored pain SM Drop-out predictors	Demographics, self-efficacy, pain catastrophising, opioid dose, comorbidities.	Early dropout associated with social stressors (*p* = 0.002/0.029, OR = 0.08/0.30); pain causal beliefs (*p* = 0.005, OR = 5.01) and pain catastrophising (*p* = 0.048, OR = 1.03) Low income significant in bivariate analysis (*p* = 0.011) only.	JBI 8/8
Kure-Beigel 2016	Denmark Urban community health centre	Mixed:Cross-sectional + qualitative	Diabetes, COPD or CVD	104	Tailored SMS Drop-out predictors	Education, age, gender, cohabitation, whether 1st meeting cancelled.	Non-completion associated with younger age (below 60) (*p* = 0.03, OR = 3.38). Non-significant trend of lower education associated with lack of completion. Qualitative study suggested comorbidity and low job control in low educated were factors.	JBI 8/8
Santorelli 2017	USA State-wide telephone survey (New Jersey)	Cross-sectional	Diabetes	4358	Any type of DSME Participation predictors	Age, sex, race, income.	42% reported attending DSME, with attendance less likely amongst lower educated (high school or less), Hispanic or ‘other’ ethnicity, those diagnosed under 2 years ago (all *p* < 0.001); the uninsured (*p* < 0.004) and those without a HCP visit for diabetes in the past year (*p* < 0.002). DSME attendance was not correlated to the number of certified DSME courses available in the area.	JBI 6/8

^1^Studies listed in order of quality as measured by Johanna Briggs Institute (JBI) criteria [30] and Sun/Oxman (S/O) subgroup analysis (for RCTs) criteria [27, 28]. RCTs listed first, followed by cohort and cross-sectional studies.

^2^Includes additional studies from the same research group where supplementary information was obtained.

**Table 4:** Effects on socioeconomic disparities: Studies examining outcomes from SM interventions, stratified by quality

Study	Theory behind intervention	Individual or group?	Intensity and duration	SES adaptions made (if any)	Demographics and SES status of population^1^	SES subgroup Comparison	Results (in terms of SES only)	Dropout by group and SES	Impact on disparity
Rothman 2004	CDSM in low SES groups is best managed by a multidisciplinary approach that is tailored to the patient’s needs and barriers.	Individual	2–4 phone or direct contacts a month (mean 38 min/month) over 12/12	Literacy adaptions, practical help to address barriers	Age: 56y mean Sex: 42%M Race: 67%EM Edu: 62% < 12 yrs. Income:74% < $20,000 Literacy: 38% ≤ 6th grade^3^	Literacy – above/below 6th grade. Correlated to education, income and insurance status.	Significant HbA1c improvement with intervention for low literacy group only; high literacy group did not differ between I/C.	Dropout low both before (study refusals) and during intervention; no difference for I/C or SES.	Reduced
De Walt 2012	People with low literacy have knowledge deficits. SMS should be adapted for their needs and provide ongoing support until mastery is achieved.	Individual	Education session + ongoing phone support for 12/12 (mean 14 calls)	Literacy adapted, intervention length varied depending on need.	Age: 60y mean Sex: 52% M Race: 61% EM^2^ Edu: 26% < 12 yr Income: 68% < $25,000 Literacy: 41%^3^ low	Literacy (S-TOFHLA). Education and subjective SES also assessed in subgroups but were weaker predictors than literacy.	Phone support more effective in low literacy group, control intervention (education session) favoured high literacy. Literacy was a stronger predictor than education/income.	Dropout equal for I/C groups and did not differ by literacy.	Reduced
Bosma 2010	SMS is focussed on increasing control and returning responsibility to the patient	Individual	2-10x1hr face-to-face sessions (mean 4) for 6/52	Extra sessions if needed	Age: 70y mean Sex: 49% M Edu: 33% primary only	Education level (primary; some high school; completed high school).	No benefit for low educated. Gains only in higher educated groups.	Increased dropout from intervention in low educated.	Increased
Moskowitz 2013	Low SES patients have more challenges with SM and need assistance with literacy, depression and social support.	Individual	0–29 phone or direct contacts (median 5) over 6/12	Patients choose own coach, language and ethnicity catered for	Age: 56y mean Sex: 49%M Race: 55% EM Edu: 36% < 12 yr	Education (less than high school; high school; some college; college degree).	Benefit for those with low medication adherence and SM ability. Education level did not affect outcome.	Dropout low both before (study refusals) and during intervention; no difference for I/C or SES.	No change
Powell 2010	SMS groups aim to motivate people to participate in their care by teaching SM skills.	Group	18x2hr over 12/12	No	Age: 63y mean Sex: 53%M Race: 40% EM Edu: 44% ≤ 12 yr Income: 52% < $30,000	Education (high school or less; above high school) and income (above/below $30000)	No improvement overall but low- income patients in intervention group had non-significant improvement on one outcome.	Dropout high both before and during intervention (in intervention group only); not reported by SES.	No change (n.s.reduction)
Smeulders 2010	The CDSMP aims to increase patient responsibility for SM by increasing self-efficacy.	Group	6 × 2.5 h over 6/52	No	Age: 67y mean Sex:72% M Edu:64% < 12 yr	Education (under or over 12 yr education).	Low educated improved more than high educated in cardiac QOL outcomes.	Dropout high before intervention (study refusals) but no difference during intervention between I/C.	Reduced
Jonker 2012	SMS works by increasing self-efficacy and improving one’s control over life and health.	Group	6 × 2.5 h over 6/52	No	Age: 82y mean Sex: 10%M Edu: 50% ≤ 9 yr	Education (over/under 9 years)	Lower educated improved on mastery (p < 0.05) but no other benefits from multiple outcomes.	Low dropout rate (but programme part of day-care centre activities).	Reduced (one outcome)
Nour 2006	Arthritis SM is achieved by Increasing knowledge and adopting health behaviours.	Individual	6-7x1hr over 8/52	No	Age: 77y mean Sex: 10%M Edu: 47% < 9 yr Perceived SES: 12% ‘financially insecure’	Education (over/under 9 years) and perceived SES	Overall minor gains, but not for those with depression or perceived low SES.	Low dropout rate	Increased
Govil 2009	SMS aims to make lifestyle changes and improve health habits.	Both	104 h over 3/12 (4 h, 2x/week)	No	Age: 60y mean Sex: 67%M Race: 5% EM Edu: 4% < 12 yr Income: 22% < $25,000	Education (high school or less; some college; college degree; postgrad degree).	All benefited equally – no difference across education levels, although lower educated had lower baseline measures.	High attendance, low dropout, unrelated to SES	No change

^1^Population SES status terms have been structured to maximise comparability between papers.

^2^*EM* ethnic minority

^3^Literacy was used as an SES measure where it was clearly correlated with education and income.

**Table 5:** Effects on socioeconomic disparities: Studies examining participation and attrition, stratified by quality

Study	Study question	Outcome	Intervention Description	SES adaptions made (if any)	SES status of population	Results (in terms of SES)	Impact on disparity
Poduval 2018	Can a DSME internet intervention engage people of differing demographics without increasing health inequity?	Use (more than 2 log-ins post registration)	Internet SM programme + email/text support and assistance to register and access site	Low literacy, developed with input from target population	Age: 58y mean Sex: 55.5%M Race: 55%EM Edu: 30% < 12 yr	No difference in use according to education. Users were reflective of the target population (inner London).	No change
Thorn 2011	Is pain SMS (CBT or education) effective in low SES groups and what are the predictors of engagement?	Initial participation and dropout	SMS groups CBT and education for 10 × 1.5 h over 10/52	Literacy adaptations and teaching	Age: 53y mean Sex: 20%M Race: 79%EM Income:86% < $30,000 Literacy score: mean 21% (50% is population mean)	Non-attendance associated with low education, literacy and income; dropout associated with low income.	Increased
Dattalo 2012	Which subgroups of multimorbid older adults are most likely to attend CDSMPs?	Completion (attend 5 or more sessions)	Stanford CDSMP 6 × 2.5 h	None	Age: 67-95 yr Sex: 43%M Race: 51.8%EM Edu: 24% < 12 yr Other SES: 42% ‘financial strain’	No effect of SES variables on course completion	No change.
Cauch-Dudek 2014	Are there disparities in utilisation of DSME soon after diagnosis?	Initial participation	Certified public health DSME programmes	Unspecified (multiple programmes)	All diabetics in Ontario, Canada diagnosed from Jan-June 2006 and followed up for 8/12.	Low SES area associated with increase in non-attendance, *p* < 0.001.	Increased
Adjei Boakye 2018	Are there are subgroups who do not participate in diabetes SM education (DSME)?	Initial participation	Diabetes SM education (DSME) - unspecified	Unspecified (multiple programmes)	Cross section of US population with diabetes	Non-participation associated with low education and low income; association stronger as education/income reduced.	Increased
Glasgow 2018	How representative of the diabetes population are those who participate or volunteer for an internet DSME study?	Initial participation	Internet DSME programme +/− support (phone calls and groups)	Available in 2 languages, no specific SES adaption	Age: 58y mean Sex: 50%M Race: 31%EM Edu: 34% ≤ 12 yrs. Income: 29% < $30,000	Higher income and education increased chance of participation, especially for self-selected people	Increased
Horrell 2017	Do those in low income areas attend CDSMPs and how can we promote higher enrolment?	Initial participation and completion	Stanford CDSMP 6 × 2.5 h	None	USA attendees of CDSMP courses Age: 58y mean 83.6% of attendees lived in the least impoverished areas.	Lowest SE area was associated with low participation (0.3% of participants) but not with low completion.	Increased (participation) No change (completion)
Hardman 2018	Do the social determinants of health affect engagement with pain SMS programmes?	Dropout (attend 3 or less sessions)	CBT-informed tailored SMS, individual or group	Programme tailored to preference/need	Age: 55y mean Sex: 42%M Income: 82% on welfare benefit Other SES: 27% ‘social stressor’	Income not significant post-regression but social stressors (substance abuse history, victim of abuse/assault) significantly associated with dropout.	Increased
Kure-Beigel 2016	Is there a social difference between those who do and don’t complete SMS programmes?	Course completion	Tailored SMS individual or group over 6–12 weeks	Programme tailored to preference/need	Age: 78% > 60 yrs. Sex: 50%M Edu: 57% < high school graduate	Education not significant post-regression but qualitative interviews suggested social factors (job/carer demands) were important.	No change - suggestive of increase
Santorelli 2017	What determines DSME participation and is it affected by the availability of DSME services?	Initial participation	DSME – unspecified type.	Unspecified (multiple programmes)	Survey sample of people living in New Jersey with diabetes	Lack of participation correlated with low education and ethnicity (*p* < 0.001) but not with income.	Increased

^1^Population SES status terms have been structured to maximise comparability between papers.

^2^*EM* ethnic minority

^3^Literacy was used as an SES measure where it was clearly correlated with education and income.

Quality analysis was undertaken using the Joanna Briggs Institute checklists [30] for randomised controlled trials (RCTs) and observational studies, and the Sun/Oxman criteria [27, 28] for subgroup analyses.

### Data synthesis

No meta-analysis was possible due to the diversity of study designs, interventions and outcome variables.

## Results

### Key study characteristics

Nineteen studies were identified, all published in English. Five studies looked at participation in SMS; five studied attrition from SMS programmes and nine assessed outcomes from SMS interventions. Interventions were very diverse, ranging from studies of the group-based Stanford Chronic Disease Self-Management Programme (CDSMP – 4 studies) to highly tailored 1–1 interventions. Table 2 and Table 3 details the main features of all studies.

### Methodological quality

Most studies were of moderate to good quality although two RCTs [39, 40] and three subgroup analyses [39–41] rated poorly. A summary of quality ratings is included in Table 2 and Table 3 and a detailed table describing how each study was assessed is available in Additional file 3.

Responses to study questions

1. Is there evidence that SES influences participation rates in SMS interventions?

Four cross-sectional studies and one cohort study looked at initial participation in SMS programmes. All were large population surveys ranging from 2600 to 80,000 people. There were three reports on diabetes SMS education programmes [42–44], one on the Stanford CDSMP [45] and the final study examined recruitment to an internet diabetes SMS programme [46]. In all studies, low SES (as measured by education, income or location) was significantly and consistently associated with lower levels of participation, suggesting that disparity in CDSM starts here. Some studies [43, 45] suggested that this imbalance was related to course availability, cost or marketing strategies. However, the studies which did match attendance to course availability and cost [42, 44] found that this did not influence participation in the low SES population. Glasgow [46] also compared participation rates in a self-selected (via media advertising) population to a referred population and found even greater disparity. As well as being of higher SES, the self-selected participants were those at lowest risk and least in need of the intervention.

There is consistent evidence that low SES is associated with lower levels of participation in SMS interventions, and some evidence that this is unrelated to access to SMS interventions.

2. Is there evidence that SES influences rates of retention or dropout from SMS interventions?

Five studies examined attrition: two cross-sectional studies and three RCTs with subgroup analysis, with sample sizes from 100 to 300. Two RCTs [41, 47] were of more advantaged populations. Of these, one reported low (22.8%) completion rates of the Stanford CDSMP [41], but predictors were related to poor physical health rather than SES. Since this was a high-risk multimorbid rather than a low SES population, dropout likely reflects increased treatment burden, as noted in other multimorbid populations [48]. The second study [47], of a diverse urban population, reported no difference in use of a supported internet programme in terms of SES (education). This intervention had been carefully tailored to maximise engagement across population groups and included extensive community involvement in the design process. Three studies [49–51] focussed on low SES populations. Two cross-sectional studies [49, 50] reported that dropout rates correlated to social stressors and lack of job flexibility, suggesting that attrition within a low SES population may be influenced by socioeconomic factors that are not captured by education or income alone. Finally, a small RCT [51] of a tailored group programme found that high levels of dropout were significantly associated with low income and education. By contrast, Horrell et al. [45] noted that although SES area predicted enrolment in the Stanford CDSMP, it did not affect rates of completion.

SES is not consistently associated with dropout from SMS interventions. SES may be one of a number of factors associated with programme attrition, as suggested by qualitative studies on this topic [52].

3. Is there evidence that SES affects clinical, behavioural or other specified outcomes following SMS interventions?

Nine studies looked at outcomes following SMS interventions, with four describing group interventions (including 2 of the Stanford CDSMP) and five individual [1] interventions. Only two of the RCTs [53, 54] were sufficiently powered for subgroup analysis and most had follow-up periods of 6 months or less.

Three of the nine studies featured outlier populations (in terms of age, sex and/or level of disadvantage), including the two lower-quality studies [39, 40] and the cohort study [55]. The findings from these studies may not be reliable or relevant to the wider low SES population.

The remaining six studies, of moderate to high quality, described broadly similar populations in terms of age, sex, education and income. Of these studies, one reported increased disparity following the intervention; two reported no change; and three studies reported a reduction in SES disparity.

Three of the studies, all individual interventions, described programmes specifically tailored for low SES groups, including extra supports and literacy adaptations. These included a 6-month peer support programme [56] and two 12-month phone support programmes [53, 57] (conducted by the same research group, but with different chronic diseases and interventions). All studies reported clinically and statistically significant changes in either hospitalisation rates [53] or HbA1c [56, 57] in favour of the intervention. Two of the studies also reported a reduction in SES disparity from the intervention, with low-literacy patients experiencing greater benefit from the intervention than their higher literacy counterparts. In an already low-SES population, this was found to be a stronger predictor than income or education. The third study (the peer support programme) reported no change in disparity, with benefits across all education levels and the greatest benefit experienced by those with poorer medication adherence and self-management ability.

The remaining studies – comprising one individual and two group interventions – did not provide specific tailoring for low SES participants. The individual intervention [58], a 6-week CBT programme designed to increase self-efficacy, found clinically significant improvements in depression only in the higher educated, with no change and higher rates of dropout in the lower educated. The group interventions, which were both for people with heart failure, included the 6-week CDSMP and a year-long SMS group programme. The CDSMP study did show short-term benefits as compared to usual care, but no overall gains at 6 or 12 months. The lower educated patients did better than their higher educated counterparts in terms of cardiac quality of life (QOL) (*p* = 0.018) over 12 months, suggesting a reduction in SES disparity, although it was not clear whether this was clinically significant. The second group programme [54] used an active education control and found no additional benefit from an SMS group. Low-income participants receiving the intervention did have a longer time to cardiac event (death or hospitalisation), but this was not statistically significant. Overall there was no change in SES disparity, nor any added benefit from the intervention.

There is limited evidence to suggest that SES does affect outcomes following SMS interventions. Interventions that were tailored for low SES participants reported significant improvements in clinical outcomes, which in some cases also included a reduction in SES disparity following the intervention.

## Discussion

### Main findings

This systematic review of disparities related to SMS interventions has reinforced observations [18–20, 22, 25] that there is a lack of research in this area. Although many studies of low SES groups have been undertaken, very few have focused on whether the outcomes compare favourably to those in higher SES groups. There are practical and statistical challenges in comparing population subgroups. Many studies had SES groupings that were fairly homogenous, limiting the ability to compare outcomes within the analysis, and almost all subgroup analyses were insufficiently powered. Larger studies and co-operation between different study populations are needed so that there is a more distinct contrast between SES levels across groups.

Responses to study questions.

1. Is there evidence that SES influences participation rates in SMS interventions?

This review confirms that low SES groups are significantly less likely to participate in SMS interventions [42–46]. Thus, healthcare disparity is increasing before an intervention even commences. In order to reach those who need the intervention, targeted recruitment and retention strategies will be needed. Self-selection runs the risk of spending limited resources on those who need them least [46].

2. Is there evidence that SES influences rates of retention or dropout from SMS interventions?

The findings in relation to retention and dropout are less clear-cut, with few studies and small sample sizes. Social factors do appear to be important [49–51], although a simple measure of SES may not capture the barriers to engagement.

3. Is there evidence that SES affects clinical, behavioural or other specified outcomes following SMS interventions?

With the limited number of high-quality studies available, there was some evidence that SES does affect outcomes following SMS interventions, depending on the type of intervention on offer. No trends were observed in terms of the SM components, which varied little between studies, or the type of service providers involved.

Programme structure (group or individual) did seem to affect both dropout rates and outcomes, with fewer benefits observed in the group interventions. In the few programmes that recorded dropout by SES, it appeared that attrition was also greater from group programmes (see Table 4 and Table 5). High rates of dropout from group programmes have been reported in several reviews of CD interventions in low SES and other vulnerable groups [21, 59], while other reviews [13, 60, 61] have noted that individually tailored interventions appear to reduce disparity. Other authors have noted that although group programmes provide beneficial social support and peer modelling [5], they can also present many barriers to a low SES population who may have less flexibility in terms of work, transport or caring demands [21, 59]. In the current review, interventions over longer time periods (6–12 months) also seemed to be more effective at reducing disparity [53, 56, 57], consistent with a CD review on similar populations [13].

### Interpretation of findings

1. ‘Low SES’ is a heterogenous group

This review suggests that SMS interventions may impact differently on low SES populations, and that more individualised treatment over longer time periods may be needed. Some writers have suggested that SES could be used as a ‘high risk’ predictor to identify those needing an earlier or more intensive intervention [23, 62], although this encompasses a large population group and has significant resource implications, emphasising the need for appropriate targeting of interventions.

Data from the current review indicates that low SES groups are heterogeneous, with additional factors such as literacy, social stressors and social capital influencing SM ability, engagement, health outcomes [49, 50, 53, 57] and thus disparity. Therefore, some low SES groups may benefit simply from better marketing of and access to generic SM courses [45] and lower-level interventions, while others will require a more intensive, tailored approach. The ability to accurately identify these groups, perhaps by using a triage instrument, could lead to more effective resource allocation, increased participation and better outcomes in terms of both efficacy and equity.

2. Are self-management mechanisms different in low SES populations?

Few studies reviewed described the theory behind the proposed SMS intervention, as noted in other reviews of SMS [12, 63], although several referred to the role of self-efficacy [40, 54, 58, 64], as described in Bandura’s social-cognitive theory [4, 5]. The studies which targeted a low SES or otherwise diverse population did note particular challenges for disadvantaged groups in terms of knowledge or literacy [47, 53, 56, 57], and those which adapted to these challenges often had better outcomes. In contrast, ‘one size fits all’ programmes [45, 46, 54, 58] had fewer benefits, and in some cases increased disparity.

SMS approaches informed only by self-efficacy have been criticised as overly individualistic [10, 11, 15] and it has been observed that the relationship between self-efficacy and self-management ability is weaker in vulnerable groups [65], indicating that other barriers play an important part. Furthermore, since the development of self-efficacy depends both on one’s behaviour and on social/environmental feedback [66], several authors [11, 58] have suggested that increasing self-efficacy may be harder if environmental feedback (e.g. job or housing insecurity) negates a belief in control over one’s circumstances.

3. What other factors are important for self-management in low SES groups?

This suggests that for SMS interventions to be effective in low SES populations, attention should be paid to other factors that influence self-management ability. Health provider/system issues [67, 68]; resources (literacy, financial, job/carer demands) [67, 69–71]; and condition demands (multimorbidity, treatment burden) [48, 71, 72] have been consistently identified in qualitative reviews as barriers to self-management. Each of these factors will impact disproportionately on a low SES population. Health providers/systems can be less accessible due to cost, literacy levels and a limited understanding of the social determinants of health by providers [67, 68]. Although few studies of SM in disadvantaged populations look at interventions at the health provider/system level [18, 21], it would seem a potentially effective way to reduce disparity without increasing the patient’s treatment burden.

Barriers related to resources and condition demands are far greater for the low SES population [73–75], who have fewer financial and social resources; higher levels of overall social complexity (job/housing insecurity, family demands, trauma history [3]); and higher rates of multimorbidity at earlier ages [76]. They experience both more disease-related workload (treatment burden) and non-disease workload (life burden) [73, 77]. Unfortunately, many SMS interventions, especially those requiring regular attendances or homework, will increase workload. Approaches that reduce patient workload or increase access to resources are rarely tried, but are likely to be important in low SES groups [73]. Phone consultations, problem-solving of specific barriers, integrating healthcare with social services and directing interventions toward healthcare practitioners rather than individual patients can all reduce treatment burden and maximise resources. Coventry [76], in a qualitative study of SM and multimorbidity, identifies three factors required for engagement in SM: capacity (resources, knowledge and energy); responsibility (shared understanding between the patient and provider about how to manage the treatment workload) and motivation. All three are negatively impacted by low SES, yet many SMS interventions [10] aim to increase motivation without recognising responsibility or capacity, and thus may contribute to increasing disparity in low SES groups.

### Strengths and limitations

This review identifies important gaps in knowledge and potential directions for future research. It reveals the assumptions informing SMS approaches and the inadequacy of using ‘low SES’ to define a population group. The study limitations include the lack of published research on disparity in SM interventions. It was difficult to conduct a comprehensive literature search of this topic because many subgroup analyses were a relatively small part of the overall paper. It is possible that some studies were missed that may have provided useful data. Meta-analysis was not possible due to the variety of studies available; therefore, no strong conclusions can be formed. In addition, the methodology of many of the studies prohibited causal inference: several studies were cross-sectional and most subgroup analyses were underpowered or did not formulate a priori hypotheses.

## Conclusion

This review has identified several important themes in relation to self-management and socioeconomic disparity. First and most obviously, there is a great need for equity considerations to be included in CD studies, as advocated by Cochrane reviewers [22, 25]. Given the strength of evidence available about social determinants of health, it should be possible to establish a priori hypotheses and sample sizes sufficient for subgroup analysis (including the availability of relevant comparator groups) for many interventions.

Secondly, any intervention in a low SES or otherwise disadvantaged group should consider its theoretical basis. Social-contextual approaches, rather than self-efficacy approaches, may be more effective. Paying greater attention to the large and consistent body of qualitative studies on barriers to SM can provide both theoretical and practical guidance as to interventions that can address disparity. Approaches such as the Cumulative Complexity Model [77], which is founded on patient burden-capacity balance, have much to offer.

Finally, levels of disadvantage vary, and there is a need for risk identification within the low SES population. For many people, improving access to simple SM interventions (e.g. assistance with childcare or transport, free programmes at community locations) may be all that is needed. For others – especially those with multimorbidity, poor literacy or social complexity – an individually tailored approach will be needed to be effective. Research to develop a risk assessment system may ensure that those most in need receive the greatest support as opposed to the current situation.

## Supplementary information

**Additional file 1.**

**Additional file 2.**

**Additional file 3.**

## Data Availability

Data sharing is not applicable to this article as no datasets were generated or analysed during the current study.

## Abbreviations

CD: Chronic disease
CDSMP: Chronic disease self-management programme
COPD: Chronic obstructive pulmonary disease
CVD: Cardiovascular disease
DSME: Diabetes self-management education
MSK: Musculoskeletal
RCTs: Randomised controlled trials
SES: Socioeconomic status
SM: Self-management
SMS: Self-management support
