# Supplementary material for: What impact do chronic disease self-management support interventions have on health inequity gaps related to socioeconomic status: a systematic review
Source: BMC Health Serv Res. 2020 Feb 27;20:150. doi: 10.1186/s12913-020-5010-4 (PMC7045733; doi:10.1186/s12913-020-5010-4)
Supplement: Supplementary file 1 — Additional file 1. [file 12913_2020_5010_MOESM1_ESM.docx]

**Systematic search terms and example of PubMed search**

**MeSH terms:** Chronic Disease Self Care or self-management Social determinants of health

**Search 1:**

'chronic disease' OR 'chronic illness' OR 'chronic condition’ OR ‘pulmonary disease' OR ‘respiratory disease’ OR 'diabetes' OR 'arthritis' OR 'chronic pain' OR ‘cardiovascular disease’ OR ‘back pain’ OR ‘musculoskeletal conditions’

**Search 2:**

'self care' OR self-care OR 'self management' OR self-management OR ‘behaviour change’ OR ‘behavior change’ OR ‘biopsychosocial rehabilitation’ OR ‘behaviour* therapy’ OR ‘behavior* therapy’

**Search 3:**

socio-economic OR socioeconomic OR equity OR inequity OR disparity OR ‘social determinants of health’ OR ‘vulnerable groups’ OR disadvantaged

**Search 4:**

outcome OR efficacy* OR participat* OR attrition OR dropout OR drop-out OR adherence OR evaluation OR access OR engage* OR attend* OR barrier OR effective* OR retention OR compliance

**MEDLINE SEARCH**

| **Search ID#** | **Search terms** | **Notes** | **Results** |
| --- | --- | --- | --- |
| Search 1 | exp Chronic disease |  | 251809 |
| S2 | “chronic disease” OR “chronic illness” OR “chronic condition” OR “pulmonary disease” OR “respiratory disease” OR diabetes OR arthritis OR “chronic pain” OR “cardiovascular disease” OR “back pain” OR “musculoskeletal condition” |  | 1243123 |
| S3 | S1 or S2 |  | 1243484 |
| S4 | exp Self Care or exp Self-Management |  | 48117 |
| S5 | “self care” OR self-care OR “self management” OR self-management OR “behaviour change” OR “behavior change” OR “biopsychosocial rehabilitation” OR “behaviour* therapy” OR “behaviour* therapy” |  | 59639 + 42364 |
| S6 | S4 or S5 |  | 99617 |
| S7 | exp "Social Determinants of Health" |  | 1762 |
| S8 | socio-economic OR socioeconomic OR equity OR inequity OR disparity OR disadvantaged OR “social determinants of health” OR “vulnerable groups” |  | 255837 |
| S9 | S7 or S8 |  | 255837 |
| S10 | outcome OR efficacy* OR participat* OR attrition OR dropout OR drop-out OR adherence OR evaluation OR access OR engage* OR attend* OR barrier OR effective* OR retention OR compliance |  | 5758281 |
| S11 | S3 and S6 and S9 and S10 |  | 805 |
| S11 | Titles reviewed |  | 805 |
| S12 | Imported |  | 100 |
| S13 | Meet sys RV criteria |  | 28 |
