# Supplementary material for: What impact do chronic disease self-management support interventions have on health inequity gaps related to socioeconomic status: a systematic review
Source: BMC Health Serv Res. 2020 Feb 27;20:150. doi: 10.1186/s12913-020-5010-4 (PMC7045733; doi:10.1186/s12913-020-5010-4)
Supplement: Supplementary file 2 — Additional file 2. [file 12913_2020_5010_MOESM2_ESM.docx]

**Table for Appendix: Articles excluded, with reasons.**

Total full-text articles reviewed: 308 (including saved articles) minus 5 duplicates = 310

Rejection reasons:

1. Not chronic disease: 22
2. Not SES: no clear reference/definition SES, focus on ethnicity exclusively: 30
3. No self-management intervention: includes reviews, commentaries, surveys of self-care or adherence without being related to a self-management intervention: 115
4. Protocol or study design/description: 32
5. Not full text or peer-reviewed (conference abstracts, unpublished dissertations): 23
6. No English translation: 2
7. Qualitative studies: 30
8. SES recorded/noted but no disparity analysis undertaken: 37

Articles kept: 19

| **Author/date** | **Rejection reason** | **Code No.** |
| --- | --- | --- |
| Ackerman 2012 | No self-management intervention | 3 |
| Adams 2010 | No clear reference/definition SES | 2 |
| Agurs-Collins 1997 | Looks at ethnicity not SES | 2 |
| Albright 2005 | Not chronic disease | 1 |
| Alter 2011 | No self-management intervention | 3 |
| Anderson 2010 | Not full-text | 5 |
| Anderson 2005 | Looks at ethnicity not SES | 2 |
| Antoniu 2003 | No clear reference/definition SES | 2 |
| Ashe 2007 | No clear reference/definition SES | 2 |
| Ausili 2016 | No self-management intervention | 3 |
| Ausili 2018 | No self-management intervention | 3 |
| Bachmann 2003 | No self-management intervention | 3 |
| Bains 2011 | No self-management intervention | 3 |
| Baird 2009 | Not chronic disease | 1 |
| Baldassari 2013 | No self-management intervention | 3 |
| Banerjee 2009 | No self-management intervention | 3 |
| Beauchamp 2014 | Not chronic disease | 1 |
| Beauchamp 2010 | Sys review - no intervention | 3 |
| Becker 2004 | No self-management intervention | 3 |
| Bennett 2018 | No analysis SES disparity | 8 |
| Benton 2018 | Looks at ethnicity not SES | 2 |
| Bergner 2017 | Protocol or design study | 4 |
| Bjarnason-Wehrens 2007 | No analysis SES disparity | 8 |
| Blackford 2017 | Not chronic disease | 1 |
| Blustein 2008 | No self-management intervention | 3 |
| Boehmer 2018 | Qualitative study | 7 |
| Boelsen-Robinson 2015 | Not chronic disease | 1 |
| Boldy 2006 | Protocol or design study | 4 |
| Borkhoff 2011 | Sys review - no intervention | 3 |
| Bos-Touwen 2015 | No self-management intervention | 3 |
| Boyd 2006 | Protocol or design study | 4 |
| Brown 2012 | Cost not SES analysis | 8 |
| Brown 2007 | No self-management intervention | 3 |
| Brown 2018 | Not chronic disease | 1 |
| Cadzow 2014 | Protocol or design study | 4 |
| Call 2016 | Qualitative study | 7 |
| Campbell 2014 | No self-management intervention | 3 |
| Campbell 2017 | No self-management intervention | 3 |
| Carnes 2013 | Protocol or design study | 4 |
| Carpenter 2017 | Protocol or design study | 4 |
| Carr 2005 | Not chronic disease | 1 |
| Chakkalakal 2015 | No self-management intervention | 3 |
| Chouinard 2013 | Protocol or design study | 4 |
| Clark 2014 | No self-management intervention | 3 |
| Conway 2017 | Qualitative study | 7 |
| Coventry 2014 | Qualitative study | 7 |
| Cramm 2011 | No self-management intervention | 3 |
| Cramm 2012 | Qualitative study | 7 |
| Crowley 2013 | No analysis SES disparity | 8 |
| Cubbin 2005 | No self-management intervention | 3 |
| David 2013 | Not chronic disease | 1 |
| Davis 2009 | No analysis SES disparity | 8 |
| De Groot 2017 | Protocol or design study | 4 |
| De Walt 2006 | Duplicate |  |
| De Walt 2006 | No analysis SES disparity | 8 |
| Dean 2015 | No clear reference/definition SES | 2 |
| De Jong 2004 | Not SM (exercise only) | 3 |
| Demonte 2015 | No self-management intervention | 3 |
| Dennis 2013 | Sys review – no intervention | 3 |
| Diaz-Toro 2015 | No self-management intervention | 3 |
| Disler 2012 | Qualitative study | 7 |
| Duenas 2018 | Protocol or design study | 4 |
| Dye 2018 | Protocol or design study | 4 |
| Dye 2018 | Duplicate |  |
| Eakin 2002 | Sys review – no intervention | 3 |
| Eakin 2010 | No analysis SES disparity | 8 |
| Edlind 2018 | No analysis SES disparity | 8 |
| Edwards 2012 | Qualitative study | 7 |
| Ell 2009 | Protocol or design study | 4 |
| Ell 2010 | No analysis SES disparity | 8 |
| Emerson 2015 | Protocol or design study | 4 |
| Ettner 2009 | No self-management intervention | 3 |
| Everson-Hock 2013 | Not chronic disease | 1 |
| Eyer 2016 | Protocol or design study | 4 |
| Feltner 2017 | No self-management intervention | 3 |
| Figaro 2009 | Qualitative study | 7 |
| Forbes 2016 | No self-management intervention | 3 |
| Foster 2008 | No self-management intervention | 3 |
| Fraser-Rodgers 2009 | No analysis SES disparity | 8 |
| Freeman 2012 | Not chronic disease | 1 |
| Fritz 2017 | Qualitative study | 7 |
| Furler 2011 | No self-management intervention | 3 |
| Gagliardino 2011 | No analysis SES disparity | 8 |
| Gallagher 2011 | No self-management intervention | 3 |
| Garmendia 2013 | Not chronic disease | 1 |
| GeBoers 2016 | No self-management intervention | 3 |
| Glazier 2006 | Sys review – no intervention | 3 |
| Goeppinger 2007 | Looks at ethnicity not SES | 2 |
| Goldman 2002 | No self-management intervention | 3 |
| Goldsmith 2014 | No self-management intervention | 3 |
| Golin 2002 | No self-management intervention | 3 |
| Gonzalez 2011 | Not full-text | 5 |
| Grande 2017 | Not full-text | 5 |
| Greene 2005 | No self-management intervention | 3 |
| Grimmer-Somers 2009 | Qualitative study | 7 |
| Guillemin 2014 | No self-management intervention | 3 |
| Hale 2010 | No self-management intervention | 3 |
| Hankonen 2009 | Not chronic disease – at risk only | 1 |
| Harley 2013 | Not chronic disease | 1 |
| Harris 2017 | Not full-text | 5 |
| Harris 2017 | Duplicate |  |
| Harris 2018 | Not full-text | 5 |
| Harris 2017 | Duplicate |  |
| Harrison 2012 | No clear reference/definition SES | 2 |
| Harvey 2007 | No self-management intervention | 3 |
| Hawe 2009 | No self-management intervention | 3 |
| Hecht 2015 | No self-management intervention | 3 |
| Heisler 2003 | No self-management intervention | 3 |
| Heltberg 2017 | No self-management intervention | 3 |
| Henderson 2014 | Qualitative study | 7 |
| Hertroijs 2016 | Not full-text | 5 |
| Hibbard 2008 | No self-management intervention | 3 |
| Higgins 2015 | Not chronic disease | 1 |
| Higgins 2018 | Not chronic disease | 1 |
| Higgs 2017 | Protocol or design study | 4 |
| Hill-Briggs 2011 | No analysis SES disparity | 8 |
| Hong 2018 | Cost not SES analysis | 8 |
| Houle 2016 | No self-management intervention | 3 |
| Hopkins 2013 | Not full-text | 5 |
| Horrell 2018 | Unpublished dissertation no peer review | 5 |
| Hughes 2016 | No analysis SES disparity | 8 |
| Humphry 1997 | Demonstration only, ethnic only | 2 |
| Jack 2012 | No self-management intervention | 3 |
| Jaramillo 2013 | No self-management intervention | 3 |
| Jeong 2018 | No analysis SES disparity | 8 |
| Jinks 2010 | Qualitative study | 7 |
| John 2007 | No clear reference/definition SES | 2 |
| Junquiera 2016 | No self-management intervention | 3 |
| Kandula 2009 | No analysis SES disparity | 8 |
| Kane 2016 | No analysis SES disparity | 8 |
| Kane 2018 | No self-management intervention | 3 |
| Kangovi 2016 | Not full-text | 5 |
| Kangvoi 2016 | No analysis SES disparity | 8 |
| Kaplan 2013 | Looks at ethnicity not SES | 2 |
| Karter 2007 | No self-management intervention | 3 |
| Keene 2018 | Qualitative study | 7 |
| Kellar 2011 | Not chronic disease | 1 |
| Kenealy 2010 | Looks at ethnicity not SES | 2 |
| Keosaian 2016 | Qualitative study | 7 |
| Kim 2016 | Sys review – no intervention | 3 |
| Kinser 2016 | No self-management intervention | 3 |
| Knight 2012 | No self-management intervention | 3 |
| Knutsen 2017 | No English translation | 6 |
| Kolbe 2002 | Not chronic disease | 1 |
| Krist 2017 | No self-management intervention | 3 |
| Laba 2013 | No self-management intervention | 3 |
| Lachance 2018 | Protocol or design study | 4 |
| LaVeist 2011 | No self-management intervention | 3 |
| Lawlor 2017 | Not full-text | 5 |
| Li 2013 | Not full-text | 5 |
| Lloyd 2006 | No self-management intervention | 3 |
| Lopez-defede 2016 | No self-management intervention | 3 |
| Lounsbury 2014 | No self-management intervention | 3 |
| Lowe 2013 | Not full-text | 5 |
| Lynch 2011 | No self-management intervention | 3 |
| Machenbach 2008 | No self-management intervention | 3 |
| Maindal 2011 | Not chronic disease | 1 |
| Maitra 2010 | No self-management intervention | 3 |
| Mao 2017 | Qualitative study | 7 |
| Margolis 2013 | Not chronic disease | 1 |
| Mayberry 2016 | Protocol or design study | 4 |
| Mayberry 2016 | No self-management intervention | 3 |
| McCarthy 2013 | Protocol or design study | 4 |
| McCollum 2009 | No self-management intervention | 3 |
| Mead 2010 | Qualitative study | 7 |
| Meland 2011 | No self-management intervention | 3 |
| Merius 2017 | Sys review – no intervention | 3 |
| Miech 2009 | No self-management intervention | 3 |
| Mills 2015 | No self-management intervention | 3 |
| Mills 2014 | Protocol or design study | 4 |
| Mishra 2011 | No self-management intervention | 3 |
| Mitchell 2012 | Looks at ethnicity not SES | 2 |
| Mohammadi 2018 | No analysis SES disparity | 8 |
| Moser 2017 | Not full-text | 5 |
| Murimi 2010 | Not chronic disease | 1 |
| Naranjo 2012 | Looks at ethnicity not SES | 2 |
| Nelson 2016 | Not full-text | 5 |
| Oh 2017 | No self-management intervention | 3 |
| Omachi 2013 | No self-management intervention | 3 |
| O’Neil 2014 | No self-management intervention | 3 |
| Osborn 2016 | No self-management intervention | 3 |
| Osborn 2013 | No self-management intervention | 3 |
| Osborn 2014 | Protocol or design study | 4 |
| Osborne 2013 | Protocol or design study | 4 |
| Packer 2012 | No analysis SES disparity | 8 |
| Pandit 2014 | No self-management intervention | 3 |
| Parker 2018 | Sys review – no intervention | 3 |
| Parsons 2017 | Qualitative study | 7 |
| Patel 2016 | No self-management intervention | 3 |
| Pavlishyn 2016 | No self-management intervention | 3 |
| Peek 2014 | No self-management intervention | 3 |
| Pesantes 2015 | Sys review – no intervention | 3 |
| Piette 2013 | No self-management intervention | 3 |
| Piper 2015 | No self-management intervention | 3 |
| Piper 2013 | No self-management intervention | 3 |
| Plaksin 2016 | Not full-text | 5 |
| Poleshuck 2010 | Protocol or design study | 4 |
| Potter 2018 | Qualitative study | 7 |
| Protheroe 2013 | Qualitative study | 7 |
| Protheroe 2016 | Protocol or design study | 4 |
| Ramal 2012 | Qualitative study | 7 |
| Rashid 2017 | Looks at ethnicity not SES | 2 |
| Rebecca Paradiso 2017 | No self-management intervention | 3 |
| Redman 2007 | No self-management intervention | 3 |
| Rendle 2013 | Qualitative study | 7 |
| Ricci-Caballo 2013 | Protocol or design study | 4 |
| Roberts 2015 | Protocol or design study | 4 |
| Rosal 2009 | Protocol or design study | 4 |
| Rosal 2011 | No analysis SES disparity | 8 |
| Rotberg 2013 | Not full-text | 5 |
| Rotberg 2014 | Not full-text | 5 |
| Rothschild 2016 | Protocol or design study | 4 |
| Ruggiero 1997 | No self-management intervention | 3 |
| Ryan 2013 | No analysis SES disparity | 8 |
| Ryvicker 2012 | No self-management intervention | 3 |
| Sajatovic 2018 | No analysis SES disparity | 8 |
| Sarkar 2006 | No self-management intervention | 3 |
| Schafer 2010 | No clear reference/definition SES | 2 |
| Schecteman 2008 | No self-management intervention | 3 |
| Schillinger 2009 | No analysis SES disparity | 8 |
| Schillinger 2006 | No self-management intervention | 3 |
| Schmiitz 2009 | No self-management intervention | 3 |
| Schoenberg 2011 | Qualitative study | 7 |
| Schulman-Green 2016 | Qualitative study | 7 |
| Schulz 2005 | Protocol or design study | 4 |
| Secrest 2011 | No self-management intervention | 3 |
| Selhy 2007 | No self-management intervention | 3 |
| Shah 2009 | No clear reference/definition SES | 2 |
| Sheridan 2015 | Qualitative study | 7 |
| Shippee 2012 | No self-management intervention | 3 |
| Shreck 2014 | No clear reference/definition SES | 2 |
| Silverman 2018 | Qualitative study | 7 |
| Sixta 2008 | No analysis SES disparity | 8 |
| Skelly 2009 | No analysis SES disparity | 8 |
| Small 2013 | No clear reference/definition SES | 2 |
| Smith 2012 | No clear reference/definition SES | 2 |
| Smith 2013 | No clear reference/definition SES | 2 |
| Smith 2010 | No self-management intervention | 3 |
| Sokol 2016 | Duplicate |  |
| Sokol 2016 | Sys review – no intervention | 3 |
| Solomon 2012 | Protocol or design study | 4 |
| Srulovici 2018 | No analysis SES disparity | 8 |
| Stafford 2012 | No self-management intervention | 3 |
| Stalker 2015 | No self-management intervention | 3 |
| Surapeni 2018 | Not full-text | 5 |
| Swavely 2014 | No analysis SES disparity | 8 |
| Tan 2015 | No self-management intervention | 3 |
| Tetra Dewi 2013 | Not chronic disease | 1 |
| Thackeray 2004 | Looks at ethnicity not SES | 2 |
| Thom 2013 | No analysis SES disparity | 8 |
| Thompson 2014 | No self-management intervention | 3 |
| Thorn 2018 | No analysis SES disparity | 8 |
| Tiliakos 2011 | Not full-text | 5 |
| Torres 2010 | No English translation | 6 |
| Torres 2107 | Not full-text | 5 |
| Trief 2013 | Looks at ethnicity not SES | 2 |
| Trief 2013 | No analysis SES disparity | 8 |
| Tucker 2014 | No analysis SES disparity | 8 |
| Vaccaro 2016 | Looks at ethnicity not SES | 2 |
| Vaccaro 2012 | Looks at ethnicity not SES | 2 |
| Van der Vlegel 2016 | Not full-text | 5 |
| Van Dyke 2013 | Not full-text | 5 |
| Van Hecke 2017 | Sys review – no intervention | 3 |
| Van Olmen 2015 | No analysis SES disparity | 8 |
| Van Scoyoc 2010 | Sys review – no intervention | 3 |
| Varming 2018 | Protocol or design study | 4 |
| Verevkina 2014 | No clear reference/definition SES | 2 |
| Verma 2017 | Limited self-management – exercise only | 3 |
| Vest 2013 | Qualitative study | 7 |
| Vijayaraghavan 2011 | No self-management intervention | 3 |
| Vimalavathini 2008 | No self-management intervention | 3 |
| Vissenberg 2017 | Qualitative study | 7 |
| Vissenberg 2017 | No analysis SES disparity | 8 |
| Von Leupoldt 2012 | No clear reference/definition SES | 2 |
| Walker 2003 | No self-management intervention | 3 |
| Walker 2010 | No self-management intervention | 3 |
| Walker 2014 | No self-management intervention | 3 |
| Walker 2014 | Protocol or design study | 4 |
| Walker 2015 | No self-management intervention | 3 |
| Walker 2015 | No self-management intervention | 3 |
| Walker 2016 | No self-management intervention | 3 |
| Wallace 2013 | No self-management intervention | 3 |
| Walters 2012 | Qualitative study | 7 |
| Walton 2012 | Protocol or design study | 4 |
| Walton-Moss 2014 | Sys review – no intervention | 3 |
| Wayne 2015 | No analysis SES disparity | 8 |
| Weaver 2014 | Qualitative study | 7 |
| Werfalli 2015 | Protocol or design study | 4 |
| White 2015 | No analysis SES disparity | 8 |
| Wilson 2017 | Not chronic disease | 1 |
| Wilson 2017 | Qualitative study | 7 |
| Wolf 2014 | No analysis SES disparity | 8 |
| Wong 2015 | No clear reference/definition SES | 2 |
| Yadav 2018 | No clear reference/definition SES | 2 |
| Yamashita 2012 | No self-management intervention | 3 |
| Young 2009 | Not full-text | 5 |
|  |  |  |
