# Supplementary material for: What impact do chronic disease self-management support interventions have on health inequity gaps related to socioeconomic status: a systematic review
Source: BMC Health Serv Res. 2020 Feb 27;20:150. doi: 10.1186/s12913-020-5010-4 (PMC7045733; doi:10.1186/s12913-020-5010-4)
Supplement: Supplementary file 3 — Additional file 3. [file 12913_2020_5010_MOESM3_ESM.docx]

**Trial Quality assessment**

Y = yes; N= no; N/A = not applicable; U = unspecified/unclear in study; score = number of criteria met.

1. **RCT studies quality assessment (Johanna Briggs Institute)**

| **Author/date** | **Correct randomisation** | **Concealed allocation** | **Similar baselines intervention/control** | **Blind participant** | **Blind assessor** | **Identical treatment** | **Follow-up complete** | **ITT analysis** | **Same outcomes** | **Outcomes reliable** | **Appropriate analysis** | **Appropriate design** | **Score** |
| --- | --- | --- | --- | --- | --- | --- | --- | --- | --- | --- | --- | --- | --- |
| Poduval 2018 | Y | Y | Y | Y | Y | Y | Y | Y | Y | Y | Y | Y | 12 |
| Thorn 2011 | Y | Y | Y | Y | Y | Y | Y | Y | Y | Y | Y | Y | 12 |
| Bosma 2011 | Y | Y | Y | N/A | Y | Y | Y | Y | Y | Y | Y | Y | 11 |
| Powell 2010 | Y | Y | Y | N/A | Y | Y | Y | Y | Y | Y | Y | Y | 11 |
| Rothman 2004 | Y | Y | Y | N/A | Y | Y | Y | Y | Y | Y | Y | Y | 11 |
| Dattalo 2012 | Y (cluster) | Y | Y | N/A | Y | Y | Y | Y | Y | Y | Y | Y | 11 |
| DeWalt 2012 | Y | Y | N (adjusted) | N/A | Y | Y | Y | Y | Y | Y | Y | Y | 10 |
| Smeulders 2010 | Y | Y | N (adjusted) | N/A | Y | Y | Y | Y | Y | Y | Y | Y | 10 |
| Moskowitz 2013 | Y | Y | N (adjusted) | N/A | Y | Y | Y | Y | Y | Y | Y | Y | 10 |
| Jonker 2012 | Y (cluster) | U | N (not adjusted) | N/A | U | Y | Y | N excluded dropouts and dementia patients | Y | U | Y | Y | 6 |
| Nour 2006 | U | U | Y | N/A | Y | U – other services also available. | U | Y | Y | U | U | Y | 5 |

1. **Subgroup analysis quality assessment (Sun/Oxman criteria)**

| **Study** | **Clinical importance** | **Statistical significance** | **A priori hypothesis** | **Few hypotheses** | **Within study comparison** | **Consistency across studies** | **Theory supported** | **Variables specified pre-randomisation** | **Apriori direction specified** | **Independent effect -not confounders** | **Consistent interaction in study** | **Score** |
| --- | --- | --- | --- | --- | --- | --- | --- | --- | --- | --- | --- | --- |
| Rothman 2004 | Y | Y | Y | Y | Y | Y | Y | Y | Y | Y | Y | 11 |
| DeWalt 2012 | Y | Y | Y | Y | Y | Y | Y | Y | Y | Y | Y | 11 |
| Poduval 2018 | Y | Y (null hypothesis supported) | Y | Y – limited variables | Y | U | Y | Y | Y | Y | Y | 10 |
| Bosma 2011 | Y | Y | Y | Y | Y | Unclear – few studies | Y | Y | N- opposite | Y | Y | 9 |
| Moskowitz 2013 | Y | Yes – p=0.02 for SM ability | N –looking for predictors | Y –regression, 1 outcome only | Y | U | Y | Y | Y | Y | Y | 9 |
| Thorn 2011 | Y | Y p=0.01 and 0.02 | N- looking for predictors | Y – regression, 1 outcome only | Y | Y | Y | Y | N | Y | Y | 9 |
| Smeulders 2010 | Y | Y – p=0.018 | N - looking for predictors | Y- regression, 1 outcome only | Y | U | Y | Y | N | Y | U | 7 |
| Powell 2010 | Y | N | N - looking for predictors | Y – regression, 1 outcome only | Y | U | Y | Y | N | Y | U | 6 |
| Dattalo 2012 | Y | Weak - one poorly validated outcome | N – looking for predictors | Y- regression, 1 outcome only | Y | N | N | Y | N | Y | U | 5 |
| Jonker 2012 | Y | Weak p<0.05 for 1 outcome only | N – looking for predictors | N - 10 variables 7 outcomes | Y | U | Y | Y | N | Y | N – only relevant to 1 outcome | 5 |
| Nour 2006 | Y | Weak – one poorly validated outcome | N – looking for predictors | No – 7 variables, 8 outcomes | Y | U | Y | Y | N | Y | N - only relevant to 1 outcome | 5 |

1. **Cross sectional studies quality assessment (Johanna Briggs Institute)**

| **Study name** | **Inclusion criteria** | **Subjects and setting** | **Exposure measurement** | **Condition measurement** | **Confounders** | **Confounder strategies** | **Outcome measurement** | **Appropriate analysis** | **Score** |
| --- | --- | --- | --- | --- | --- | --- | --- | --- | --- |
| Adjae Boakye 2018 | Y | Y | Y | Y | Y | Y | Y | Y | 8 |
| Glasgow 2018 | Y | Y | Y | Y | Y | Y | Y | Y | 8 |
| Hardman 2018 | Y | Y | Y | Y | Y | Y | Y | Y | 8 |
| Horrell 2017 | Y | Y | Y | Y | Y | Y | Y | Y | 8 |
| Kure-Beigel 2016 | Y | Y | Y | Y | Y | Y | Y | Y | 8 |
| Santorelli 2017 | Y | Y | Y | Y | Y | U | Y | U | 6 |

1. **Cohort studies quality assessment (Johanna Briggs Institute)**

| **Study** | **Similar groups** | **Exposure details** | **Exposure measurement** | **Confounders** | **Confounding strategies** | **Outcome freedom** | **Outcomes measured** | **Follow-up time** | **Follow-up complete** | **Follow-up strategies** | **Appropriate analysis** | **Score** |
| --- | --- | --- | --- | --- | --- | --- | --- | --- | --- | --- | --- | --- |
| Cauch-Dudek 2014 | Y | Y | Y | Y | Y | Y | Y | Y- 8 months | Y | N/A | Y | 10 |
| Govil 2009 | N | Y | Y | Y | Y | Y | Y | N- immediate post-intervention only | Y | Y | Y | 9 |
